# P-1626. Balancing Priorities: The Role of Infection Prevention Departments in Acute Care Facilities During the COVID-19 Pandemic

**DOI:** 10.1093/ofid/ofaf695.1802

**Published:** 2026-01-11

**Authors:** Tara Schmidt, Patrick Moeller, Monika Pogorzelska-Maziarz, Lauren N Satchell

**Affiliations:** Villanova University, Villanova, Pennsylvania; Thomas Jefferson University, Philadelphia, Pennsylvania; Villanova University, Villanova, Pennsylvania; Villanova University, Villanova, Pennsylvania

## Abstract

**Background:**

COVID-19 required acute care facilities to reallocate resources and adapt to rapidly changing circumstances. This shift resulted in diversion of resources away from routine infection prevention and control (IPC) activities. The long-term effects of this remain poorly understood, particularly in the context of balancing pandemic response with routine IPC priorities. Therefore, we aimed to assess how hospitals prioritized IPC activities during the pandemic.

**Methods:**

Between August and December 2023, we conducted an electronic survey of all hospitals participating in the National Healthcare Safety Network. We collected data on the role of IPC departments and the prioritization of activities. The data were analyzed using descriptive statistics.

**Results:**

854 IPC departments participated in the survey. Approximately 90% of participants reported leading or supporting pandemic response-related activities during the first two years of the pandemic (2020-2021). The majority noted the following activities were high priority: attending COVID-19 related operations/incident command meetings and providing IPC input on COVID-19 related protocols (95%), updating and providing education/support for personal protective equipment (PPE) donning/doffing practices and supporting changes in practices as new guidelines emerged (91%), patient-to-patient and employe-to-patient contact tracing (78%) and rounding to support staff and provide consultation (75%). Activities most frequently reported to be set aside or put on hold included: participating in non-IPC related environment of care rounds (51%), performance improvement teams unrelated to emergency 47%), attendance at committee meetings (43%), observational audits (41%), routine policy and procedure review (39%), and surveillance activities for lower-risk, lower-impact healthcare-associated infections (33%). Activities that were most frequently reported as delegated to non-IP staff were PPE counts (57%), vaccine clinic staffing (45%), and employee-to-employee contact tracing (30%).

**Conclusion:**

The results showed that IPC departments were pivotal in spearheading and supporting numerous high-impact COVID-19 response activities, all while managing ongoing surveillance and other priority IPC activities.

**Disclosures:**

All Authors: No reported disclosures

